# Comparison of the TNM9th and 8th editions for localized and locally advanced anal squamous cell carcinoma treated nonsurgically and proposal of a new stage grouping system

**DOI:** 10.1002/cam4.70119

**Published:** 2024-08-19

**Authors:** Aihong Zheng, Yiwen Wang, Shuang Li, Yingjie Wang, Hong'en Xu, Jieni Ding, Bingchen Chen, Tao Song, Lei Lai

**Affiliations:** ^1^ Cancer Center, Department of Medical Oncology Zhejiang Provincial People's Hospital, Affiliated People's Hospital, Hangzhou Medical College Hangzhou People's Republic of China; ^2^ Department of Clinical Medical Engineering The Second Affiliated Hospital, Zhejiang University School of Medicine Hangzhou People's Republic of China; ^3^ Cancer Center, Department of Radiation Oncology Zhejiang Provincial People's Hospital, Affiliated People's Hospital, Hangzhou Medical College Hangzhou People's Republic of China; ^4^ Cancer Center, Department of Radiation Oncology Zhejiang Provincial People's Hospital, Jinzhou Medical University Jinzhou People's Republic of China; ^5^ Department of Oncology Tongxiang First People's Hospital Jiaxing People's Republic of China; ^6^ Department of General Surgery, Cancer Center, Division of Colorectal Surgery Zhejiang Provincial People's Hospital, Affiliated People's Hospital, Hangzhou Medical College Hangzhou People's Republic of China

**Keywords:** anal squamous cell carcinoma, comparison, overall survival, TNM

## Abstract

**Objective:**

To compare the survival discrimination of the TNM9th and 8th editions for localized and locally advanced anal squamous cell carcinoma (ASCC) treated nonsurgically and suggest a simple revised staging system with data from the Surveillance, Epidemiology, and End Results (SEER) database.

**Methods:**

Overall survival (OS) was the primary endpoint. Survival comparisons between the T and N stages and the different staging systems were performed using the Kaplan–Meier method and log‐rank test, followed by correlation analysis and variable importance analysis (VIA). Additionally, multivariate analysis was employed to identify significant predictors, which were further visualized using a nomogram. Finally, calibration curve, C‐index, and decision curve analysis (DCA) were applied to assess the performance of the different staging systems.

**Results:**

A total of 5384 patients with ASCC were analyzed, revealing superior discrimination OS by the TNM9th edition compared to that by the TNM8th edition. Multivariate analysis identified the T and N stages as significant OS predictors (all *p* < 0.001). However, ambiguity persisted in stage III subgroups within the TNM9th edition, showing OS times of 102 months for stage IIIA disease, 88 months for stage IIIB disease, and 128 months for stage IIIC disease (all *p* > 0.05). Correlation analysis demonstrated an increased correlation for the T stage between the TNM8th and 9th editions (*ρ* value from 0.7 to 0.89), while the N stage correlation decreased (*ρ* value from 0.84 to 0.56). VIA and the prognostic nomogram highlighted the greater importance of the T stage over the N stage. Based on these findings, a new staging system was developed, and its clinical utility was confirmed through calibration curves, C‐index values (from 0.598 to 0.604), and DCAs.

**Conclusions:**

Our new staging system exhibited slightly better prognostic value compared to the TNM9th staging systems for nonmetastatic ASCC and warrants further validation.

## 1 INTRODUCTION

Anal carcinoma is a relatively uncommon neoplasm originating around the anal tissue and accounts for 2.8% of gastrointestinal tract cancers.^1^, ^2^, ^3^ The GLOBOCAN 2020 study reported >50,000 new cases of anal carcinoma, with nearly 20,000 related deaths occurring worldwide in 2020.^4^ Anal squamous cell carcinoma (ASCC) is the predominant type of anal carcinoma among all histological subtypes.^5^ Until the research by Nigro et al., surgical resection was the historical curative treatment option.^6^ Since that study publication, the paradigm for treating localized and locally advanced ASCC has shifted to external beam radiation therapy (EBRT) combined with concurrent chemotherapy (CTx),^7^, ^8^ along with local excision only for a small proportion of patients with early‐stage ASCC without high‐risk factors.^9^ The long‐term follow‐up results of the landmark Radiation Therapy Oncology Group (RTOG) 0529 trial also confirmed the survival benefits of concurrent chemoradiotherapy (CCRT), revealing an overall survival (OS) rate of 76% and 68% at the 5‐ and 8‐year follow‐ups, respectively.^10^ However, the degree of positive survival outcomes for patients with localized and locally advanced ASCC treated by CCRT can vary depending on several factors, particularly the cancer stage.

The classical American Joint Committee on Cancer (AJCC) tumor‐node‐metastasis (TNM) system is a widely accepted staging system in oncology. The TNM system helps to decide between different treatment options according to the disease stage, enabling satisfactory prognostic prediction in daily clinical practice.^11^, ^12^ Over the last nearly two decades, the staging system for ASCC has been updated from the AJCC TNM6th edition to the latest 9th edition.^13^, ^14^, ^15^ Compared with the TNM6th and 7th editions, the TNM8th and 9th editions do not have any changes in the definition of T classification, whereas major revisions are present in the N definition. In particular, the involvement of the external iliac lymph nodes (LNs) is considered a site of regional disease in the TNM8th and 9th editions, but it was classified as a site of distant metastasis in the previous editions. Furthermore, in the case of patients diagnosed using TNM8th staging, previous data from the National Cancer Database (NCDB) indicated that patients with stage IIIA anal cancer (T1‐2N1M0) had a better prognosis than those diagnosed with stage IIB anal cancer (T3N0M0). This finding is a paradox to the common understanding that higher staging is associated with a worse prognosis. Thus, the TNM9th staging system introduced certain adjustments, including (1) the redefinition of stage IIB as T1‐2N1M0 disease, (2) the redefinition of stage IIIA as T3N0–1 M0 disease, and (3) the elimination of stage 0 disease.^15^ However, this update has not yet been confirmed in other cohorts.

In this study, we first validated the clinical usefulness of the TNM9th staging system for patients with localized and locally advanced ASCC who underwent nonsurgical treatment. Further, we compared the efficacy of the TNM9th and 8th staging systems. Finally, a simple staging system was suggested for patients with localized and locally advanced ASCC based on data acquired from the Surveillance, Epidemiology, and End Results (SEER) database.

## 2 PATIENTS AND METHODS

### 2.1 Patient selection

This retrospective study retrieved data spanning 2004 to 2018 from the SEER 18 population‐based registries (SEER* Stat 8.3.6), which cover nearly half of the United States population,^16^ using the International Classification of Diseases for Oncology, 3rd edition (ICD‐O‐3) site code of C21.0–9. The following inclusion criteria were used in recruiting the patients for this study: (1) a confirmed histopathological diagnosis of ASCC; (2) primary diagnosis of nonmetastatic ASCC; (3) age ≥18 years; and (4) a clear indication that the ASCC was treated nonsurgically. The major exclusion criteria were as follows: (1) more than one primary cancer indicated in the registry; (2) treatment other than EBRT ± CTx; and (3) ambiguous data about the TNM stage and survival time <1 month. The Institutional Review Board (IRB) of Zhejiang Provincial People's Hospital determined that the data in this dataset contained no personal identifiers and were publicly available after permission. Therefore, a formal IRB review was waived.

### 2.2 Data processing

In the initial database, tumors were registered based on the AJCC TNM6th edition for patients with ASCC diagnosed between 2004 and 2015, the SEER combined stage for those diagnosed in 2016 and 2017, and the Derived extent of disease (EOD) 2018 T and N (2018+) for those diagnosed after 2018. The definitions for the stages diagnosed after 2016 are available at https://seer.cancer.gov/seerstat/variables/seer/ajcc‐stage/. Patients were then restaged according to the stage categories of the TNM8th and 9th editions. Furthermore, in the TNM6th edition and patients diagnosed in 2016 and 2017, the involvement of the external iliac LNs was defined as distant metastasis. This aspect could not be identified and further processed from the original dataset. Sixty years was set as the cutoff point for age comparison according to the results of another SEER study.^17^ The diagnosis period was categorized as a ternary factor and divided into 2004–2008, 2009–2013, and 2014–2018. SEER registry was defined as a binary factor and segregated as California and NonCalifornia.^18^ Other variables were processed as described in our previous studies.^19^, ^20^, ^21^

### 2.3 Statistical analysis

Survival data, including survival status (alive or dead) and survival time in months, were also obtained from the database. OS was determined as the duration between the day of ASCC diagnosis and the day of death due to any reason or the last follow‐up day recorded in the database. Baseline characteristics of the patients were summarized using descriptive statistics and frequency tables. Survival curves were plotted using the Kaplan–Meier method and log‐rank test. A higher likelihood ratio chi‐square value in the Cox regression model indicated better homogeneity in the staging discrimination.^22^ Correlation between the T and N stages and the different tumor stages and the importance of the two variables (T and N stages within the different staging systems) were analyzed using the Spearman rank correlation coefficient (*ρ*) and random forest algorithm with the “corrplot” and “randomForestSRC” R packages. The Cox proportional hazard regression model was employed to evaluate the prognostic variables in multivariate analysis using the significant factors identified by univariate analysis (expressed via hazard ratio [HR] and 95% confidence interval [CI]). Furthermore, a prognostic nomogram for predicting 5‐ and 10‐year OS was constructed based on the multivariate analysis. Lastly, the different staging systems were evaluated using Harrell's concordance index (C‐index) values, calibration curves, and decision curve analyses (DCAs) as described in our previous study.^20^

Statistical analyses were performed via the Statistical Package for the Social Sciences software (version 25.0; IBM Corporation, Armonk, NY, USA) and R software (version 3.6.2; https://www.r‐project.org, Institute for Statistics and Mathematics, Vienna, Austria). Survival curves were plotted using GraphPad Prism 8.0 (GraphPad Software, San Diego, CA, USA). A two‐sided statistical significance level of *p* < 0.05 was employed.

## 3 RESULTS

### 3.1 Patient characteristics

A total of 5384 eligible patients with ASCC registered from 2004 to 2018 and who underwent nonsurgical treatment were included in the final analysis. Table 1 presents the baseline characteristics of the included patients. The median age at diagnosis was 59 years, and 3742 patients were female (69.5%). Overall, more than half of the patients (54.6%) were diagnosed with T2, while positive LNs were detected in 36.8%. In the TNM8th staging system, the number of patients with stage I (T1N0M0), IIA (T2N0M0), IIB (T3N0M0), IIIA (T1‐2N1M0), IIIB (T4N0M0), and IIIC (T3‐4N1M0) disease were 771 (14.3%), 1966 (36.5%), 572 (10.6%), 1167 (21.7%), 91 (1.7%), and 817 (15.2%), respectively. In the TNM9th staging system, the number of patients with stage I (T1N0M0), IIA (T2N0M0), IIB (T1‐2N1M0), IIIA (T3N0–1 M0), IIIB (T4N0M0), and IIIC (T4N1M0) disease were 771 (14.3%), 1966 (36.5%), 1167 (21.7%), 1256 (23.3%), 91 (1.7%), and 133 (2.5%), respectively.

**TABLE 1:** Baseline clinicopathological and treatment features of patients with localized and locally advanced anal squamous cell carcinoma who underwent nonsurgical treatment.

Characteristic	Frequency (%)
Age at diagnosis (years)	
Median (IQR)	59 (53–69)
≤60	2742 (50.9)
>60	2642 (49.1)
Sex	
Female	3742 (69.5)
Male	1642 (30.5)
Marital status	
Married	2199 (40.8)
Unmarried and others	3185 (59.2)
Diagnosis period	
2004–2008	1004 (18.6)
2009–2013	1797 (33.4)
2014–2018	2583 (48.0)
Race	
White	4692 (87.1)
Nonwhite	692 (12.9)
Differentiation	
Well or fairly differentiated	2202 (40.9)
Poorly or undifferentiated	1601 (29.7)
Unknown	1581 (29.4)
Registry site	
California	2282 (42.4)
NonCalifornia	3102 (57.6)
T stage	
T_0‐1_	965 (17.9)
T_2_	2939 (54.6)
T_3_	1256 (23.3)
T_4_	224 (4.2)
N stage	
Negative	3400 (63.2)
Positive	1984 (36.8)
AJCC TNM8th	
I	771 (14.3)
IIA	1966 (36.5)
IIB	572 (10.6)
IIIA	1167 (21.7)
IIIB	91 (1.7)
IIIC	817 (15.2)
AJCC TNM9th	
I	771 (14.3)
IIA	1966 (36.5)
IIB	1167 (21.7)
IIIA	1256 (23.3)
IIIB	91 (1.7)
IIIC	133 (2.5)
Proposal	
I	965 (17.9)
IIA	1966 (36.5)
IIB	973 (18.1)
IIIA	1256 (23.3)
IIIB	224 (4.2)
EBRT	
No/unknown	261 (4.8)
Yes	5123 (95.2)
CTx	
No/unknown	462 (8.6)
Yes	4922 (91.4)

Abbreviations: AJCC, American Joint Committee on Cancer; CTx, chemotherapy; EBRT, external beam radiation therapy; IQR, interquartile range; TNM, Tumor‐Node‐Metastasis.

### 3.2 Survival outcomes using the TNM8th and 9th editions

**FIGURE 1:**
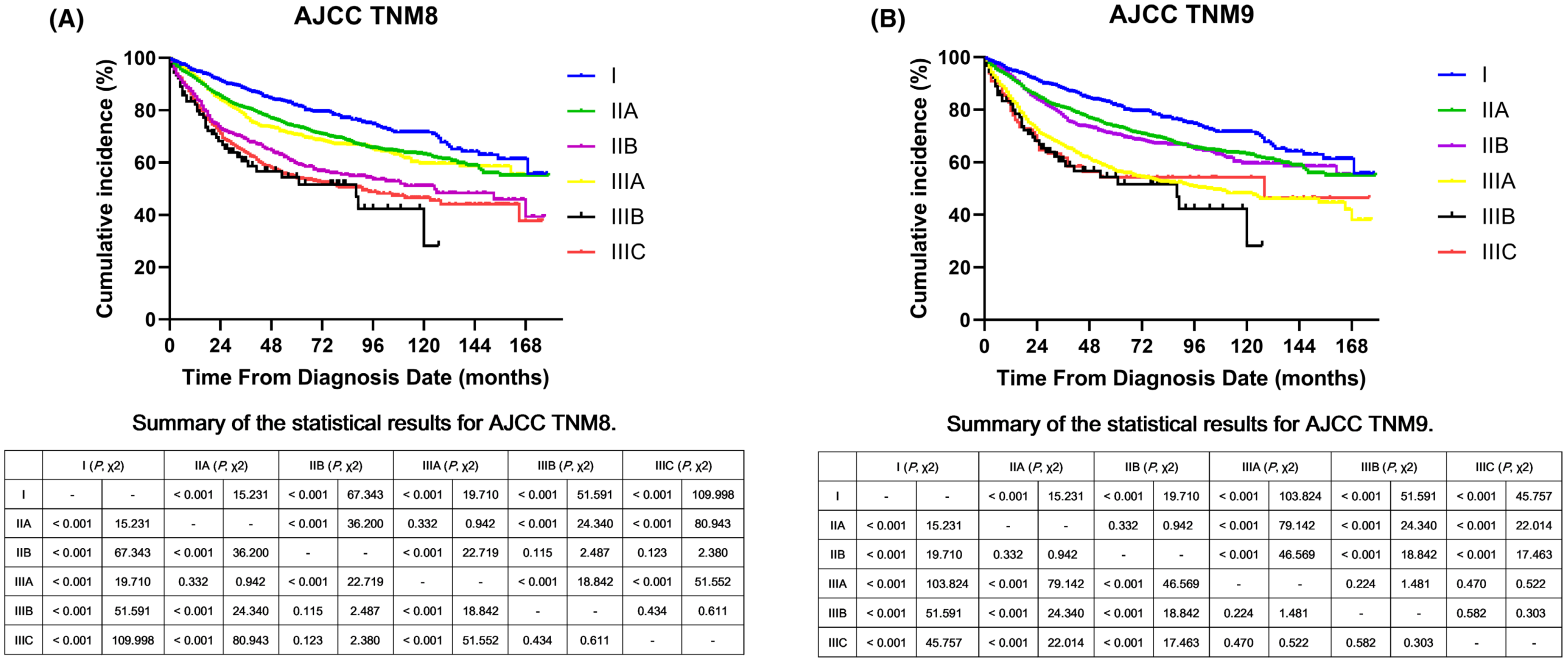
Overall survival (OS) of patients with localized and locally advanced anal squamous cell carcinoma who received nonsurgical treatment according to (A) the AJCC TNM8th and (B) TNM9th staging systems.

Figure 1 illustrates the survival curves based on the different staging groups with detailed statistical results. Overall, the 5‐ and 10‐year OS rates were 69.7% (95% CI, 0.683–0.711) and 59.7% (95% CI, 0.557–0.617), respectively, and the median OS time was not reached at the last follow‐up.

Briefly, in the AJCC TNM8th edition, a paradox was observed between stage IIB and IIIA diseases (median OS: 125 months for stage IIB disease vs. not reached for stage IIIA disease), with this difference exhibiting statistical significance (*p* < 0.001; Figure 1A). Furthermore, no statistical difference was identified in OS between stage IIIB and IIIC diseases (median OS: 88 months for stage IIIB disease vs. 91 months for stage IIIC disease, *p* = 0.434). In the updated AJCC TNM9th edition (Figure 1B), improved discrimination with a significant difference was observed between stage IIB and IIIA diseases (median OS: not reached for stage IIB disease vs. 102 months for stage IIIA disease; *p* < 0.001). However, certain ambiguity was still demonstrated among the subgroups of stage III diseases (i.e., 102 months for stage IIIA disease vs. 88 months for stage IIIB disease vs. 128 months for stage IIIC disease), although these findings were not significant. Thus, we aimed to improve the survival discrimination further using the AJCC TNM9th edition.

### 3.3 Analysis of T and N stages

**FIGURE 2:**
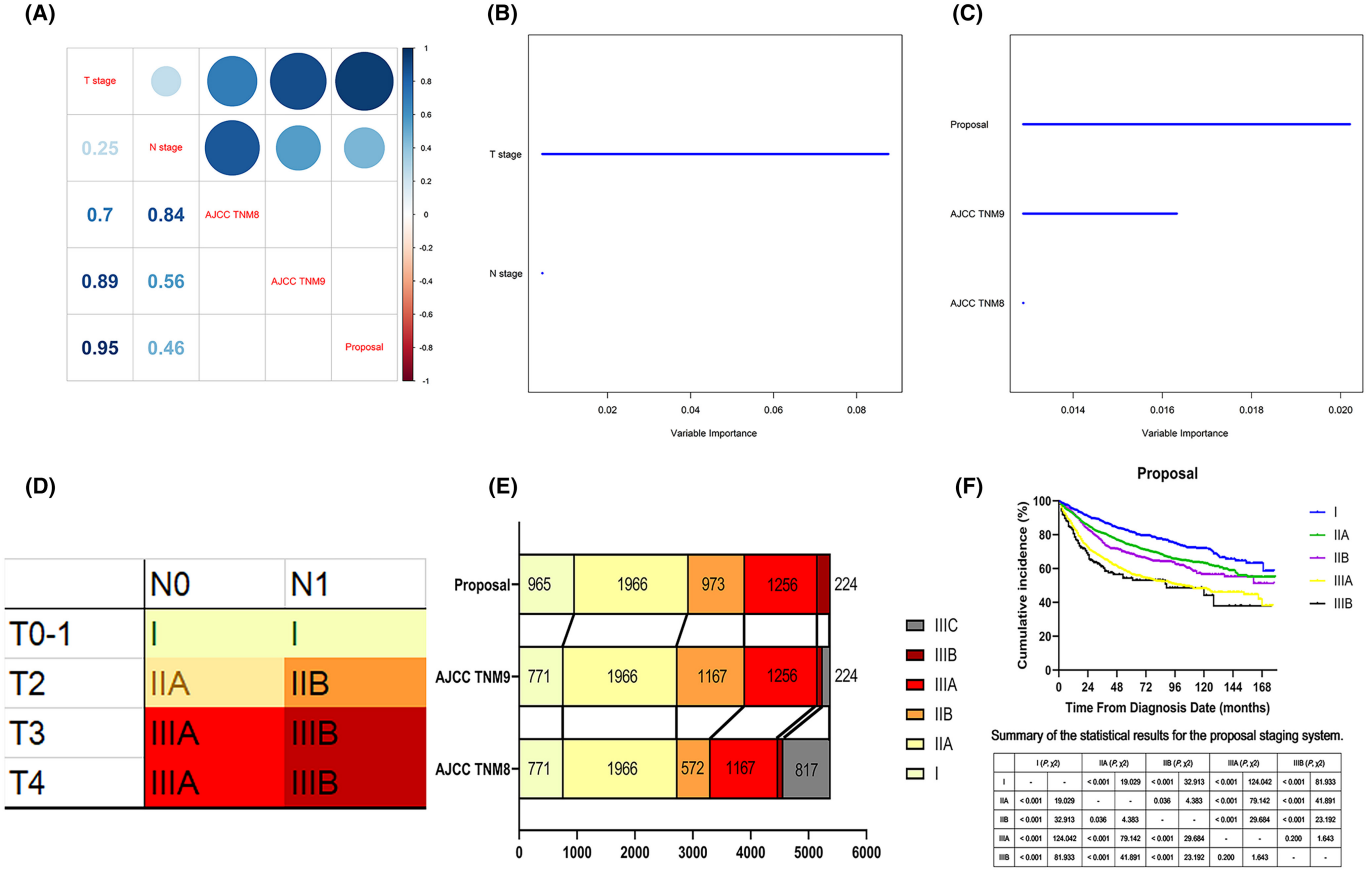
(A) Correlation coefficients between the T and N stages and the three different staging systems. (B) Variable importance analysis between the T and N stages in the enrolled patients. (C) Variable importance analysis of the TNM8th and TNM9th staging and the proposed staging systems in the included patients. (D) The proposed staging system for patients with localized and locally advanced anal squamous cell carcinoma who received nonsurgical treatment. (E) The proportion of patients diagnosed using the three different staging systems. (F) OS analysis of the enrolled patients based on the proposed staging system.

**FIGURE 3:**
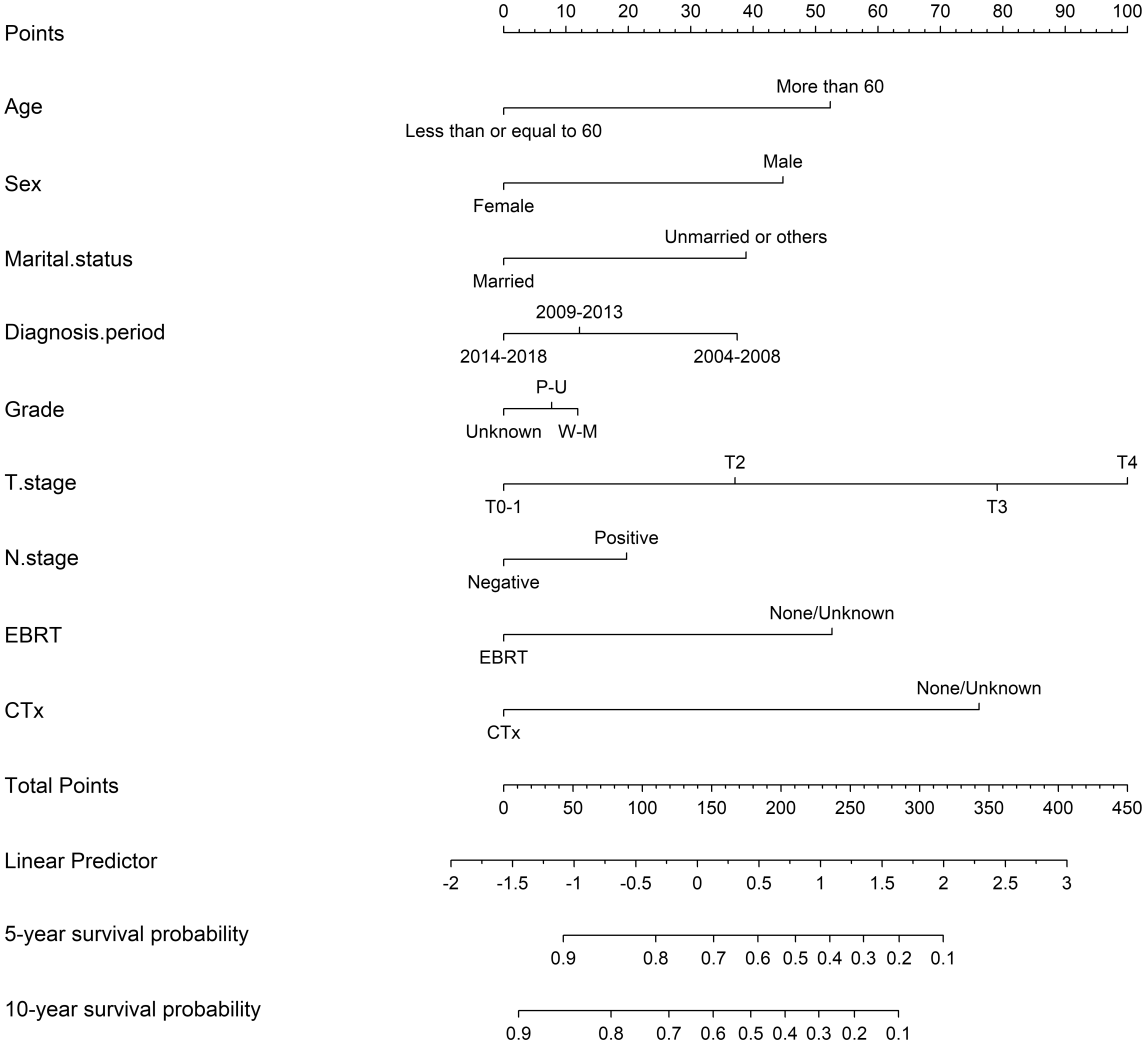
A nomogram predicting the OS of the patients with ASCC according to the multivariate analysis.

Initially, we performed a correlation analysis between the T and N stages and the TNM8th and 9th staging systems (Figure 2A). The analysis revealed an increased correlation (*ρ* value from 0.7 to 0.89) for the T stage in the TNM8th and 9th staging systems, respectively. In contrast, the N stage showed a decreased correlation in the TNM8th and 9th staging systems. Next, variable importance analysis (VIA) was conducted by applying a random forest algorithm to the enrolled patients (Figure 2B). Compared with the N stage, the T stage had a more prominent prognostic influence on OS (the relative importance rate [RIR] for the N stage was 5%, whereas the RIR for the T stage was 100%). A similar result was also obtained when VIA was conducted between the TNM8th and 9th staging systems (Figure 2C). Furthermore, univariate and multivariate Cox regression analyses were performed to identify predictive factors for OS (Table S1). We abandoned the different staging systems in the multivariate Cox regression model due to the multicollinearities between the T and N stages and the staging systems (Figure 2A). Ultimately, a nomogram based on the multivariate Cox regression model results was constructed and evaluated (Figure 3). The T stage was revealed as the most important prognostic parameter for OS estimation, whereas the N stage was the penultimate component in the nomogram.

### 3.4 Proposal of a simple stage grouping system

Based on the above‐mentioned results and the increasing number of patients with early‐stage anal cancer receiving definitive treatments, the continuous prognostic importance of the T stage was emphasized. A simplified staging system, without modifications to the T and N stage definitions, was proposed as depicted in Figure 2D. In this system, patients with T1 disease were categorized as stage I (T1N0–1 M0), irrespective of regional LN status. T2 disease was classified as stage II, with patients exhibiting negative LNs classified as stage IIA (T2N0M0) and those exhibiting positive LNs as stage IIB (T2N1M0). T3–4 diseases were defined as stage III, with patients exhibiting negative LNs classified as stage IIIA (T3‐4N0M0) and those exhibiting positive LNs as stage IIIB (T3‐4N1M0). A tighter correlation for the T stage was observed in the proposed system compared to the TNM8th and 9th systems (Figure 2A). The proposed system also placed greater importance on OS in the random forest algorithm (Figure 2C). Figure 2E demonstrates the distribution of patients enrolled based on different staging systems. The proposed system introduced a major change for 194 patients with T1N1M0 ASCC, downstaging them from stage IIB in the TNM9th system to stage I. Additionally, T4N0–1 M0 patients (stage IIIB–C in the TNM9th staging) were uniformly classified as stage IIIB in the proposed staging system. A survival curve based on the proposed system is shown in Figure 2E. Except for the nonsignificant survival benefit of stage IIIA over stage IIIB patients (median OS: 102 months vs. 89 months; *p* = 0.200), other group comparisons showed significant differences (*p* < 0.05).

### 3.5 Comparison and validation of the proposed staging system

**FIGURE 4:**
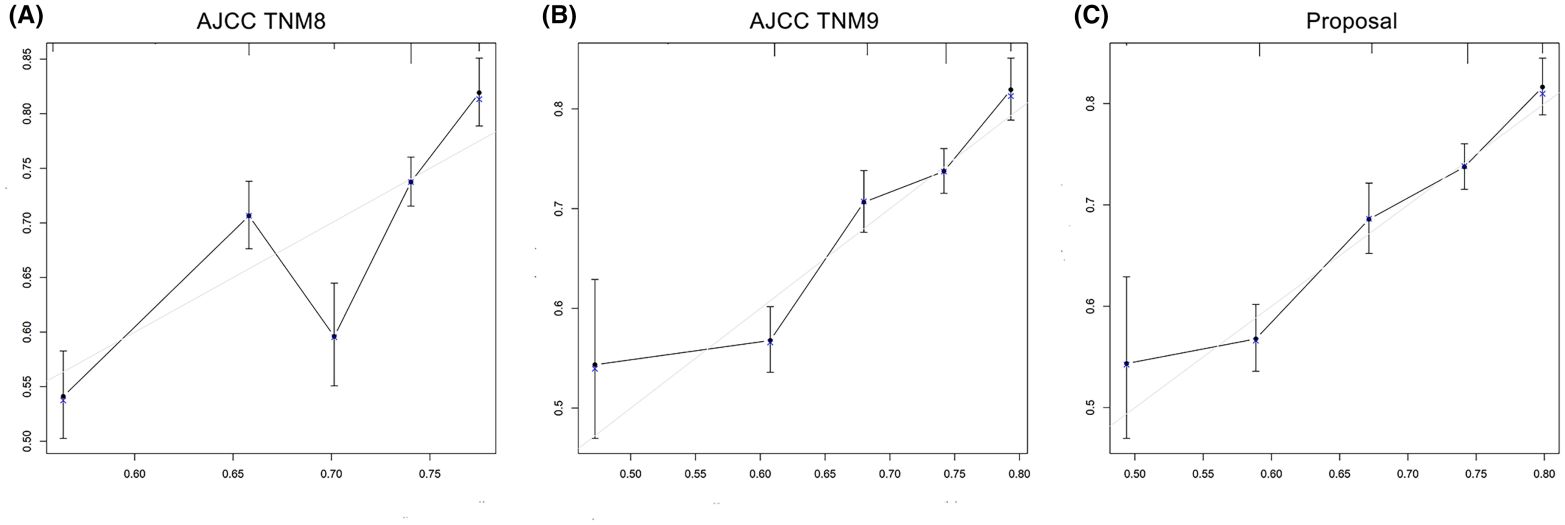
Calibration curves for predicting the 5‐year OS of the patients based on the three different staging systems. (A) TNM8th staging system. (B) TNM9th staging system. (C) The proposed staging system.

**FIGURE 5:**
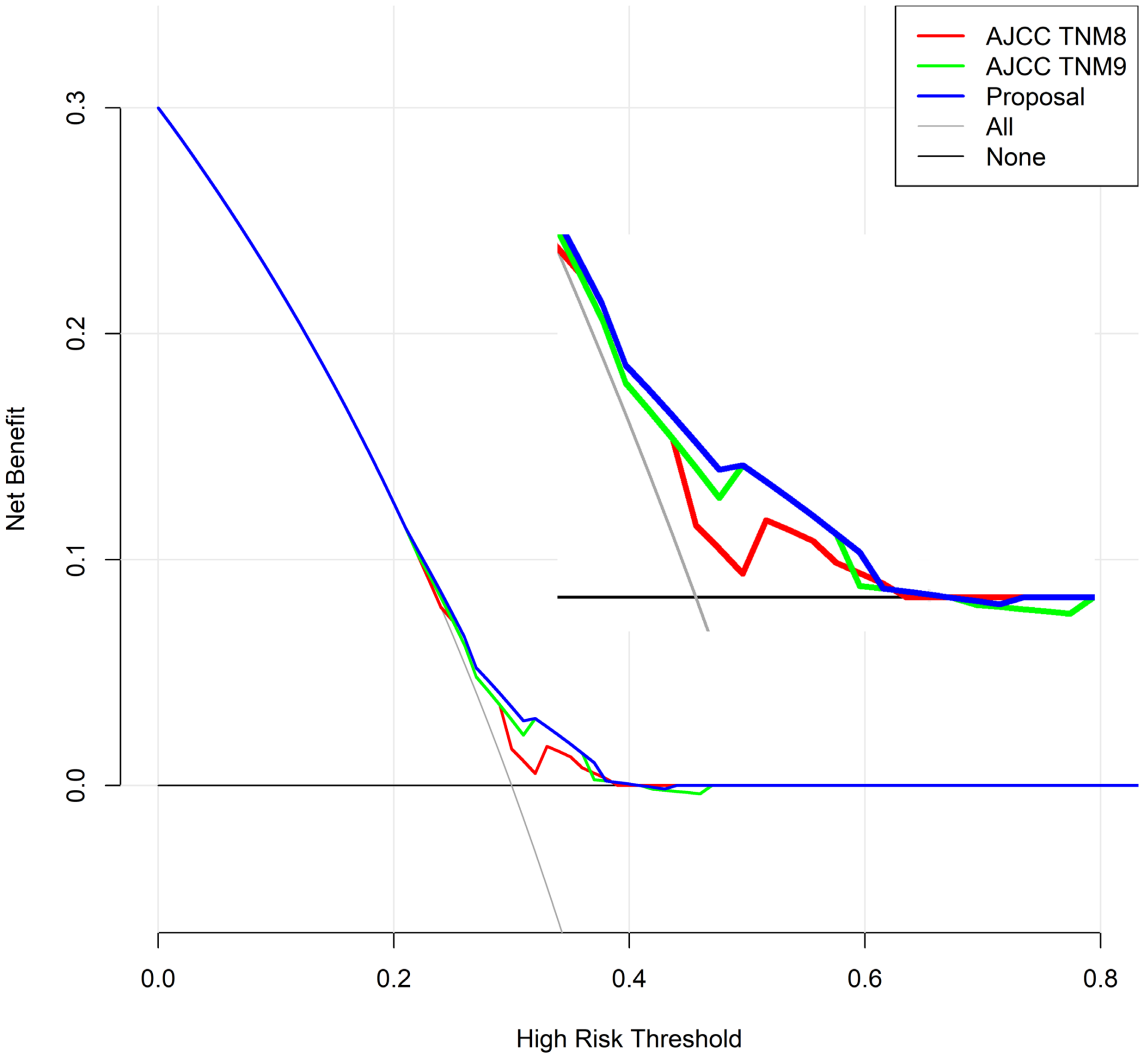
Decision curve analysis of the enrolled patients according to the three different staging systems.

The C‐index values of the TNM8th and 9th staging and the proposed staging systems for predicting 5‐year OS were 0.586, 0.598, and 0.604, respectively. The calibration curves indicated that the proposed staging system had the smoothest trend and the most satisfactory agreement between the prediction and actual survival in our patient population (Figure 4). The DCAs also suggested that the proposed staging system gained the most benefits for OS prediction within all threshold probabilities and provided greater positive net benefit than the “all” or “none” strategies for the enrolled patients (Figure 5).

### 3.6 Subgroup analysis for ASCC patients diagnosed between 2014 and 2018

The TNM9th anal cancer staging system, based on NCDB data from 2014 to 2018,^15^ prompted a subgroup analysis for ASCC patients in the SEER database from the same period, involving 2583 patients. The proposed staging system categorized 416 (16.1%) patients with stage I (T1N0–1 M0), 890 (34.5%) with IIA (T2N0M0), 501 (19.4%) with IIB (T2N1M0), 646 (25.0%) with IIIA (T3‐4N0M0), and 130 (5.0%) with IIIB (T3‐4N1M0) diseases. Figure S2 presents survival curves based on different staging systems with detailed statistical results. Due to limited follow‐up time, no significant differences in discriminative ability emerged within stage II and III subgroups between the TNM9th and proposed staging systems. However, the C‐index values for predicting 3‐year OS were 0.593, 0.600, and 0.602 for the TNM8th, TNM9th, and proposed systems, respectively. The proposed system showed the smoothest trend and best agreement between predicted and actual survival in calibration curves (Figure S3). Subgroup DCAs reconfirmed the proposed system's slight net benefit over the TNM8th and TNM9th systems, consistent with the C‐index outcomes (Figure S4).

## 4 DISCUSSION

In this study, correlation analysis and VIA between the T and N stages and the two AJCC TNM staging systems demonstrated unsatisfactory survival outcomes at certain sub‐stages, particularly stage III ASCC. Therefore, we investigated whether we could improve the prognostic usefulness of the widely accepted TNM system for patients with nonmetastatic ASCC.

After reviewing the updates from the TNM6th to 9th staging systems, major changes focused on regional LNs were observed. For example, the N2 and N3 categories in the older TNM6th and 7th staging systems based on the locations of positive LNs were removed, and new definitions of N1a, N1b, and N1c were provided in the later editions. Clinical stages (stages I–III) were then generated by integrating the T stage and the status of positive regional LNs for localized and locally advanced ASCC.^13^, ^14^, ^15^ Based on the two main guidelines on the delineation of treatment volumes for EBRT,^23^, ^24^ the drainage area of regional LNs, including the inguinal, mesorectal, superior rectal, internal iliac, obturator (N1a), and external iliac (N1b) LNs, were recommended for irradiation except in selected patients with early stage, node‐negative ASCC. In the contemporary RTOG 0529 trial, a prescribed radiotherapy (RT) dose of 50.4–54 Gy via intensity‐modulated RT (IMRT) was administered in patients with positive LNs, whereas an RT dose of 42 Gy was provided to those with negative LNs. Moreover, long‐term follow‐up analysis demonstrated a cumulative incidence rate of 16% for local‐regional failure (LRF) at the 5‐ and 8‐year follow‐ups. Additionally, distant metastasis rates of 16% and 22% were reported at the 5‐ and 8‐year follow‐ups, respectively, indicating appropriate RT dosing in regional LNs.^10^ Furthermore, advanced EBRT technologies, such as volumetric arc therapy (VMAT), helical tomotherapy (HT), and proton therapy, have facilitated satisfactory coverage of the RT doses not only to primary tumors but also to regional LNs.^25^, ^26^ A retrospective study by Gleeson et al. compared the dosimetric distributions of VMAT and HT for the definitive treatment of localized and locally advanced ASCC. The study results showed that the two treatment modalities achieved high‐quality RT plans, with HT exhibiting advantages in the dose distribution to the small bowel and bladder. However, the VMAT modality was associated with a significantly shorter delivery time than HT.^27^

The emphasis on the prognostic effect of regional LN (N stage) and not the original tumor size (T stage) might be attributed to the findings of a series of post hoc analyses of prior prospective trials. For example, data from the RTOG 9811 trial demonstrated that positive LNs were an independent prognostic factor for decreased OS and disease‐free survival (DFS), while tumor diameter only showed a significant effect on DFS.^28^ Similarly, in the ACT‐I trial conducted in the UK, positive LNs were indicated as a prognostic indicator for significantly higher LRF, tumor‐specific death, and lower OS.^29^ In the European Organization for Research and Treatment of Cancer 22,861 trial, clinically positive LNs not only significantly predicted poor OS but also forecasted decreased loco‐regional control.^30^ In our study, the multivariate Cox regression analysis supported the prognostic value of regional LNs in the enrolled patients, consistent with the previous results. However, the T stage was revealed as the most important prognostic factor for OS in our study. A similar finding supporting the prognostic effectiveness of the T stage was reported from the NCDB data analysis mentioned earlier, which included 12,968 cases of anal cancer.^15^ Correspondingly, another retrospective cohort study from France involving 82 patients with ASCC who underwent EBRT ± CTx indicated that the T stage was an independent prognostic variable for progression‐free survival (*p* = 0.04).^31^ A 2021 SEER database study reported that tumor size had a significant prognostic value for OS in 2458 patients with stage I–IV anal cancer. However, the cutoff value of the tumor size was not as per the T stage definition in the TNM staging manual.^17^

Considering that most patients with localized and locally advanced ASCC would be recommended EBRT ± CTx, the clinical stage (and not the pathological stage) is the primary factor in clinical decision‐making. Thus, accurate T staging based on the longest tumor diameter obtained on radiological imaging is crucial.^32^ Furthermore, compared with abdominal computed tomography (CT), pelvic magnetic resonance imaging (MRI) employing different scanning sequences on one of the three conventional planes is considered advantageous for clinical T categorization,^33^, ^34^ whereas the detection of metastatic regional LNs remains controversial in the current literature. As reported in previous studies, proper criteria for detecting metastatic LNs solely based on the size illustrated on a short axis might be prone to error,^34^, ^35^, ^36^ accompanied by the potential partial visualization of the inguinal nodes and missing detection of metastatic nodes due to the improper placement of the saturation band used in abdominal MRI.^32^ In this field, positron emission tomography (PET/CT) has been shown to offer superior diagnostic performance for metastatic LNs compared to CT and MRI. A previous meta‐analysis revealed that PET/CT resulted in a nodal staging change in 28% (95% CI, 0.18–0.38) of ASCC patients, while the TNM stage was altered in 41%.^37^ Additionally, PET/MRI has shown benefits for anal cancer with molecular imaging advantages and excellent soft tissue contrast resolution.^38^

This study has several limitations that need to be considered. Firstly, the present study was a retrospective analysis with patients enrolled over a wide time period (2004–2018), highlighting the need for further prospective validation with larger sample sizes. Moreover, the proposed staging system was specifically designed for localized and locally advanced ASCC patients who underwent nonsurgical treatment and was solely based on data from the SEER database, indicating the necessity for expanded confirmation across all histological types of anal cancer and integration with metastatic disease staging in external cohorts. Secondly, certain crucial parameters, including the human papillomavirus (HPV) infection status, metastasis to the external iliac LNs in patients registered between 2004 and 2017, initial tumor staging using advanced imaging techniques like MRI, PET/CT and PET/MRI, performance status, tumor marker levels, nutritional status, and treatment failure pattern, were not available in the current SEER database. Lastly, advancing treatment options for ASCC patients undergoing CCRT have introduced unknown treatment‐related variables, including different EBRT patterns (traditional two‐dimensional RT or advanced techniques like IMRT or VMAT), concurrent CTx regimens including mitomycin C and 5‐ fluorouracil or capecitabine, as well as immunotherapies, potentially introducing bias in the final inferences. To date, a de‐escalation treatment strategy has been applied for patients having head and neck SCC with positive HPV status; thus, the application of this treatment approach for ASCC is worth future investigation.

## 5 CONCLUSIONS

This study observed improved prognostic performance of the AJCC TNM9th edition over the 8th edition for patients with localized and locally advanced ASCC undergoing nonsurgical treatment. Detailed comparative analyses between the T and N stages and the TNM staging systems led to the proposal of a revised staging system that emphasizes the continuous prognostic value of the T stage. Subsequent subgroup analysis of patients diagnosed from 2014 to 2018, along with calibration curves, C‐index values, DCAs, further confirmed the superiority of the proposed staging system over the TNM9th staging system. Given the critical importance of accurate cancer staging in oncology, large‐scale validation of this new staging system for patients with ASCC is recommended in future studies.

## AUTHOR CONTRIBUTIONS

**Aihong Zheng:** Conceptualization (lead); data curation (equal); investigation (equal); writing – original draft (equal); writing – review and editing (equal). **Yiwen Wang:** Data curation (equal); formal analysis (equal); methodology (equal); software (equal); supervision (equal); validation (equal); writing – original draft (equal); writing – review and editing (equal). **Shuang Li:** Conceptualization (equal); data curation (equal); methodology (equal); writing – original draft (equal); writing – review and editing (equal). **Yingjie Wang:** Data curation (supporting); formal analysis (supporting); software (supporting); writing – original draft (equal); writing – review and editing (equal). **Hong'en Xu:** Conceptualization (equal); formal analysis (equal); validation (equal); writing – original draft (equal); writing – review and editing (equal). **Jieni Ding:** Formal analysis (equal); methodology (supporting); software (supporting); writing – original draft (equal); writing – review and editing (equal). **Bingchen Chen:** Conceptualization (supporting); methodology (supporting); resources (supporting); validation (supporting); writing – original draft (supporting); writing – review and editing (supporting). **Tao Song:** Conceptualization (lead); data curation (lead); formal analysis (lead); validation (equal); visualization (equal); writing – original draft (equal); writing – review and editing (equal). **Lei Lai:** Conceptualization (lead); formal analysis (lead); investigation (equal); methodology (equal); validation (lead); writing – original draft (equal); writing – review and editing (equal).

## FUNDING INFORMATION

This work was funded by a Grant from the Medical Science and Technology Project of Zhejiang Province, China (No. 2023KY050).

## CONFLICT OF INTEREST STATEMENT

The authors declare that they have no competing interests.

## ETHICS STATEMENT

It was not necessary to get written informed consent for participating in the present research as the information contained in the SEER database has been de‐identified and is publically available following authorization. The present research was exempted from ethical assessment by the Institutional Review Board of Zhejiang Provincial People's Hospital. We hereby certify that the present research was conducted in conformity with the Declaration of Helsinki.

## Supporting information


Figure S1.

Figure S2.

Figure S3.

Figure S4.

Table S1.

## Data Availability

The datasets used and analyzed are included in the article. Further inquiries can be directed to the corresponding author on reasonable request.

## References

[1] Wilkinson JR , Morris EJ , Downing A , et al. The rising incidence of anal cancer in England 1990‐2010: a population‐based study. Colorectal DisColorectal Dis. 2014;16:234‐239. 10.1111/codi.12553 24410872

[2] Eng C , Ciombor KK , Cho M , et al. Anal cancer: emerging standards in a rare disease. J Clin OncolJ Clin Oncol. 2022;40:2774‐2788. 35649196 10.1200/JCO.21.02566

[3] Siegel RL , Miller KD , Wagle NS , Jemal A . Cancer statistics, 2023. CA Cancer J ClinCA Cancer J Clin. 2023;73:17‐48. 10.3322/caac.21763 36633525

[4] Sung H , Ferlay J , Siegel RL , et al. Global cancer statistics 2020: GLOBOCAN estimates of incidence and mortality worldwide for 36 cancers in 185 countries. CA Cancer J ClinCA Cancer J Clin. 2021;71:209‐249. 10.3322/caac.21660 33538338

[5] Gordon PH . Squamous‐cell carcinoma of the anal canal. Surg Clin North AmSurg Clin North Am. 1988;68:1391‐1399. 10.1016/s0039-6109(16)44694-3 3057665

[6] Nigro ND , Vaitkevicius VK , Considine B Jr . Combined therapy for cancer of the anal canal: a preliminary report. 1974. Dis Colon RectumDis Colon Rectum. 1993;36:709‐711. 10.1007/BF02238600 8348857

[7] Houlihan OA , O'Neill BD . Chemoradiotherapy for anal squamous cell carcinoma. SurgeonSurgeon. 2016;14:202‐212. 10.1016/j.surge.2016.03.006 27118047

[8] Gardner IH , Watson KM . Diagnosis and treatment of anal squamous cell carcinoma. Dis Colon RectumDis Colon Rectum. 2020;63:1358‐1361. 10.1097/DCR.0000000000001791 32969877

[9] O'Brien SJ , Ellis CT , McDowell J , Galandiuk S , Polk HC Jr . Anal squamous cell carcinoma incidentally found at hemorrhoidectomy. SurgerySurgery. 2021;169:610‐616. 10.1016/j.surg.2020.08.026 33004218

[10] Kachnic LA , Winter KA , Myerson RJ , et al. Long‐term outcomes of NRG oncology/RTOG 0529: a phase 2 evaluation of dose‐painted intensity modulated radiation therapy in combination with 5‐fluorouracil and Mitomycin‐C for the reduction of acute morbidity in Anal Canal cancer. Int J Radiat Oncol Biol PhysInt J Radiat Oncol Biol Phys. 2022;112:146‐157. 10.1016/j.ijrobp.2021.08.008 PMC8688291 34400269

[11] Edge SB , Compton CC . The American joint committee on cancer: the 7th edition of the AJCC cancer staging manual and the future of TNM. Ann Surg OncolAnn Surg Oncol. 2010;17:1471‐1474. 10.1245/s10434-010-0985-4 20180029

[12] Amin MB , Greene FL , Edge SB , et al. The eighth edition AJCC cancer staging manual: continuing to build a bridge from a population‐based to a more "personalized" approach to cancer staging. CA Cancer J ClinCA Cancer J Clin. 2017;67:93‐99. 10.3322/caac.21388 28094848

[13] Greene FL . TNM staging for malignancies of the digestive tract: 2003 changes and beyond. Semin Surg OncolSemin Surg Oncol. 2003;21:23‐29. 10.1002/ssu.10018 12923913

[14] Dahl O , Myklebust MP , Dale JE , et al. Evaluation of the stage classification of anal cancer by the TNM 8th version versus the TNM 7th version. Acta OncolActa Oncol. 2020;59:1016‐1023. 10.1080/0284186X.2020.1778180 32574087

[15] Janczewski LM , Faski J , Nelson H , et al. Survival outcomes used to generate version 9 American joint committee on cancer staging system for anal cancer. CA Cancer J ClinCA Cancer J Clin. 2023;73:516‐523. 10.3322/caac.21780 37114458

[16] Ren X , Huang T , Tang X , et al. Development and validation of nomogram models to predict radiotherapy or chemotherapy benefit in stage III/IV gastric adenocarcinoma with surgery. Front OncolFront Oncol. 2023;13:1223857. 10.3389/fonc.2023.1223857 PMC10466399 37655111

[17] Tang J , Zhu L , Huang Y , et al. Development and validation of prognostic survival nomograms for patients with Anal Canal cancer: a SEER‐based study. Int J Gen Med. 2021;14:10065‐10081. 34984027 10.2147/IJGM.S346381 PMC8709559

[18] Amirian ES , Fickey PA Jr , Scheurer ME , Chiao EY . Anal cancer incidence and survival: comparing the greater san‐Francisco bay area to other SEER cancer registries. PLoS OnePLoS One. 2013;8:e58919. 10.1371/journal.pone.0058919 PMC3590168 23484057

[19] Chen F , Chen L , Zhang Y , Shi L , Xu H , Song T . Survival comparison between squamous cell carcinoma and adenocarcinoma for radiotherapy‐treated patients with stage IIB‐IVA cervical cancer. Front OncolFront Oncol. 2022;12:895122. 10.3389/fonc.2022.895122 PMC9352995 35936684

[20] Yu W , Lu Y , Shou H , et al. A 5‐year survival status prognosis of nonmetastatic cervical cancer patients through machine learning algorithms. Cancer MedCancer Med. 2023;12:6867‐6876. 10.1002/cam4.5477 PMC10067071 36479910

[21] Shou H , Wan Q , Xu H , Shi L , Song T . Stage IIB‐IVA cervix carcinoma in elderly patients treated with radiation therapy: a longitudinal cohort study by propensity score matching analysis. BMC Womens HealthBMC Womens Health. 2023;23:270. 10.1186/s12905-023-02427-8 PMC10193738 37198594

[22] Chen MW , Yen HH . Comparison of the sixth, seventh, and eighth editions of the American joint committee on cancer tumor‐node‐metastasis staging system for gastric cancer: a single institution experience. Medicine (Baltimore). 2021;100:e27358. 34596145 10.1097/MD.0000000000027358 PMC8483861

[23] Myerson RJ , Garofalo MC , El Naqa I , et al. Elective clinical target volumes for conformal therapy in anorectal cancer: a radiation therapy oncology group consensus panel contouring atlas. Int J Radiat Oncol Biol PhysInt J Radiat Oncol Biol Phys. 2009;74:824‐830. 10.1016/j.ijrobp.2008.08.070 PMC2709288 19117696

[24] Ng M , Leong T , Chander S , et al. Australasian gastrointestinal trials group (AGITG) contouring atlas and planning guidelines for intensity‐modulated radiotherapy in anal cancer. Int J Radiat Oncol Biol PhysInt J Radiat Oncol Biol Phys. 2012;83:1455‐1462. 10.1016/j.ijrobp.2011.12.058 22401917

[25] Meyer J , Czito B , Yin FF , Willett C . Advanced radiation therapy technologies in the treatment of rectal and anal cancer: intensity‐modulated photon therapy and proton therapy. Clin Colorectal CancerClin Colorectal Cancer. 2007;6:348‐356. 17311699 10.3816/CCC.2007.n.003

[26] Shridhar R , Shibata D , Chan E , Thomas CR . Anal cancer: current standards in care and recent changes in practice. CA Cancer J ClinCA Cancer J Clin. 2015;65:139‐162. 10.3322/caac.21259 25582527

[27] Gleeson I , Rose C , Spurrell J . Dosimetric comparison of helical tomotherapy and VMAT for anal cancer: a single institutional experience. Med DosimMed Dosim. 2019;44:e32‐e38. 10.1016/j.meddos.2018.12.004 30639142

[28] Gunderson LL , Winter KA , Ajani JA , et al. Long‐term update of US GI intergroup RTOG 98‐11 phase III trial for anal carcinoma: survival, relapse, and colostomy failure with concurrent chemoradiation involving fluorouracil/mitomycin versus fluorouracil/cisplatin. J Clin OncolJ Clin Oncol. 2012;30:4344‐4351. 23150707 10.1200/JCO.2012.43.8085 PMC3515768

[29] Glynne‐Jones R , Sebag‐Montefiore D , Adams R , et al. Prognostic factors for recurrence and survival in anal cancer: generating hypotheses from the mature outcomes of the first United Kingdom coordinating committee on cancer research anal cancer trial (ACT I). CancerCancer. 2013;119:748‐755. 10.1002/cncr.27825 23011911

[30] Bartelink H , Roelofsen F , Eschwege F , et al. Concomitant radiotherapy and chemotherapy is superior to radiotherapy alone in the treatment of locally advanced anal cancer: results of a phase III randomized trial of the European Organization for Research and Treatment of cancer radiotherapy and gastrointestinal cooperative groups. J Clin OncolJ Clin Oncol. 1997;15:2040‐2049. 9164216 10.1200/JCO.1997.15.5.2040

[31] Gauthe M , Richard‐Molard M , Rigault E , et al. Prognostic value of serum CYFRA 21‐1 1 in patients with anal canal squamous cell carcinoma treated with radio(chemo)therapy. BMC CancerBMC Cancer. 2018;18:417. 29653564 10.1186/s12885-018-4335-4 PMC5899349

[32] Golia Pernicka JS , Sheedy SP , Ernst RD , Minsky BD , Ganeshan D , Rauch GM . MR staging of anal cancer: what the radiologist needs to know. Abdom Radiol (NY). 2019;44:3726‐3739. 31041496 10.1007/s00261-019-02020-4

[33] Granata V , Fusco R , Reginelli A , et al. Radiological assessment of anal cancer: an overview and update. Infect Agent Cancer. 2016;11:52. 27752279 10.1186/s13027-016-0100-y PMC5062854

[34] Ciombor KK , Ernst RD , Brown G . Diagnosis and diagnostic imaging of Anal Canal cancer. Surg Oncol Clin N AmSurg Oncol Clin N Am. 2017;26:45‐55. 10.1016/j.soc.2016.07.002 PMC10174401 27889036

[35] Parikh J , Shaw A , Grant LA , et al. Anal carcinomas: the role of endoanal ultrasound and magnetic resonance imaging in staging, response evaluation and follow‐up. Eur RadiolEur Radiol. 2011;21:776‐785. 10.1007/s00330-010-1980-7 20890758

[36] Durot C , Dohan A , Boudiaf M , Servois V , Soyer P , Hoeffel C . Cancer of the Anal Canal: diagnosis, staging and follow‐up with MRI. Korean J RadiolKorean J Radiol. 2017;18:946‐956. 10.3348/kjr.2017.18.6.946 PMC5639160 29089827

[37] Jones M , Hruby G , Solomon M , Rutherford N , Martin J . The role of FDG‐PET in the initial staging and response assessment of anal cancer: a systematic review and meta‐analysis. Ann Surg OncolAnn Surg Oncol. 2015;22:3574‐3581. 10.1245/s10434-015-4391-9 25652048

[38] Jayaprakasam VS , Ince S , Suman G , et al. PET/MRI in colorectal and anal cancers: an update. Abdom Radiol (NY). 2023;48:3558‐3583. 37062021 10.1007/s00261-023-03897-y

