# Supplementary figures and images for: Comparison of the TNM9th and 8th editions for localized and locally advanced anal squamous cell carcinoma treated nonsurgically and proposal of a new stage grouping system
Source: Cancer Med. 2024 Aug 19;13(16):e70119. doi: 10.1002/cam4.70119 (PMC11331335; doi:10.1002/cam4.70119)

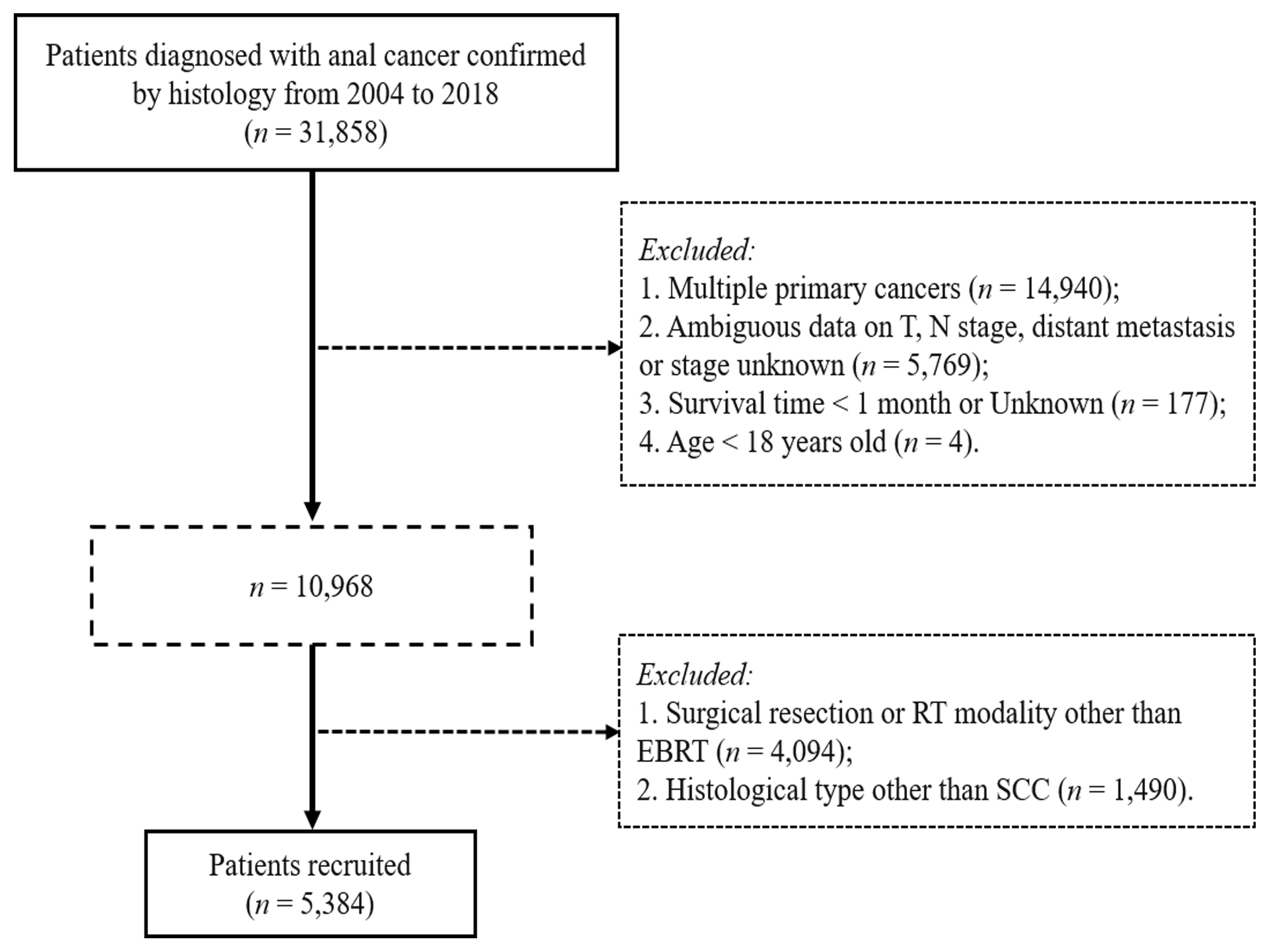

Supplement: Supplementary file 1 — Figure S1. [file CAM4-13-e70119-s005.tif]

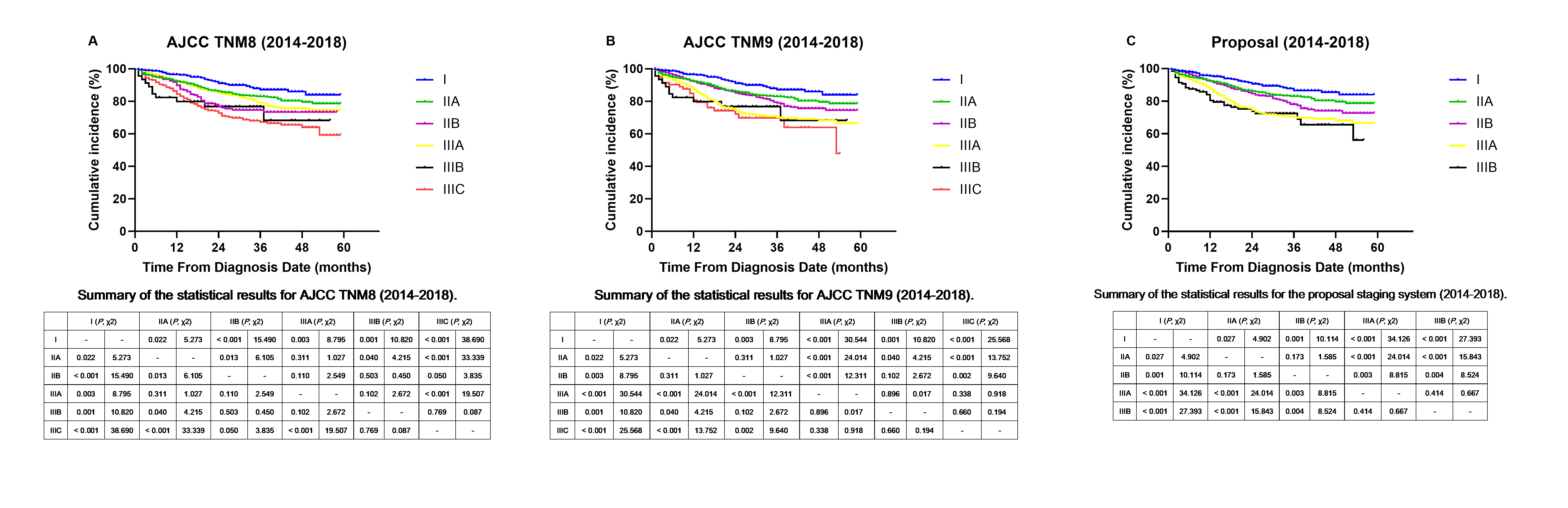

Supplement: Supplementary file 2 — Figure S2. [file CAM4-13-e70119-s004.tif]

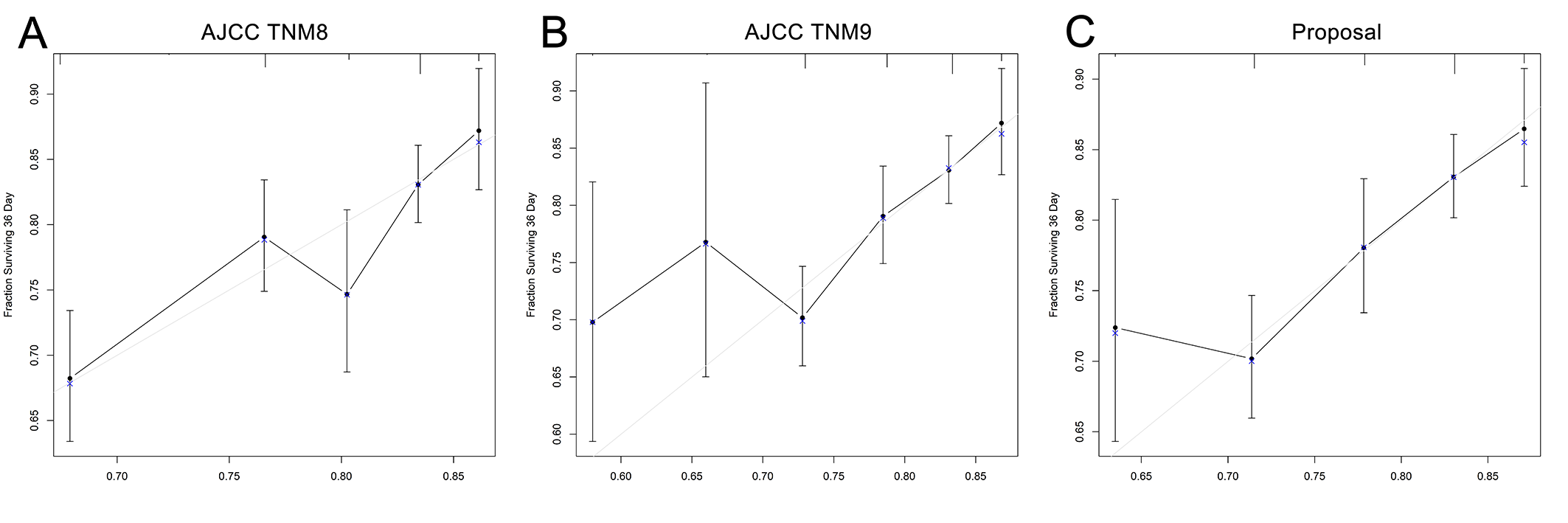

Supplement: Supplementary file 3 — Figure S3. [file CAM4-13-e70119-s002.tif]

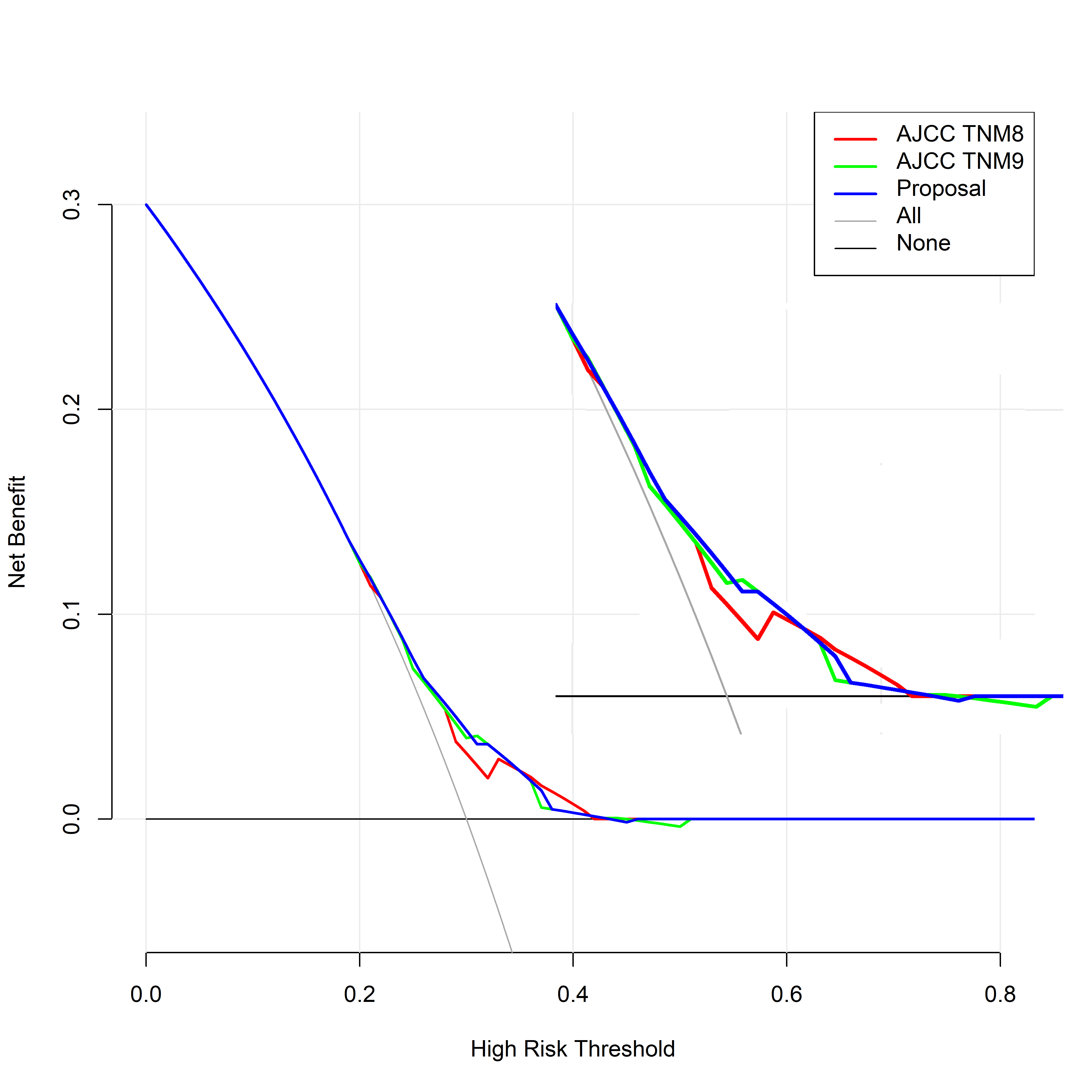

Supplement: Supplementary file 4 — Figure S4. [file CAM4-13-e70119-s003.tif]
