# Supplementary material for: Comparison of the TNM9th and 8th editions for localized and locally advanced anal squamous cell carcinoma treated nonsurgically and proposal of a new stage grouping system
Source: Cancer Med. 2024 Aug 19;13(16):e70119. doi: 10.1002/cam4.70119 (PMC11331335; doi:10.1002/cam4.70119)
Supplement: Supplementary file 5 — Table S1. [file CAM4-13-e70119-s001.docx]

Table S1. Univariate and multivariate analyses of the OS of patients with localized and locally advanced anal squamous cell carcinoma who received non-surgical treatment.

|  | Overall survival (OS) | | | | | | | | |
| --- | --- | --- | --- | --- | --- | --- | --- | --- | --- |
|  | Univariate | | | |  | Multivariate | | | |
| *Factor* | ***P*** *value* | *HR* | *95% CI Lower* | *95% CI Upper* |  | ***P*** *value* | *HR* | *95% CI Lower* | *95% CI Upper* |
| *Age, ≤ 60 vs. > 60* | < 0.001 | 1.714 | 1.548 | 1.898 |  | < 0.001 | 1.807 | 1.627 | 2.008 |
| *Race, White vs. Non-white* | 0.006 | 1.218 | 1.058 | 1.403 |  | 0.342 | 1.072 | 0.929 | 1.238 |
| *Sex, Female vs. Male* | < 0.001 | 1.663 | 1.500 | 1.843 |  | < 0.001 | 1.648 | 1.483 | 1.831 |
| *Marital status, Married vs. Unmarried and others* | < 0.001 | 1.793 | 1.606 | 2.002 |  | < 0.001 | 1.537 | 1.374 | 1.719 |
| *Diagnosis period, reference: 2004-2008* | 1.000 |  |  |  |  | 1.000 |  |  |  |
| 2009-2013 | < 0.001 | 0.800 | 0.707 | 0.905 |  | < 0.001 | 0.754 | 0.665 | 0.854 |
| 2014-2018 | < 0.001 | 0.748 | 0.650 | 0.860 |  | < 0.001 | 0.659 | 0.572 | 0.760 |
| *Grade, reference: W-F* | 1.000 |  |  |  |  | 1.000 |  |  |  |
| P-U | 0.033 | 0.878 | 0.779 | 0.990 |  | 0.446 | 0.954 | 0.845 | 1.077 |
| Unknown | 0.006 | 0.841 | 0.743 | 0.951 |  | 0.032 | 0.874 | 0.772 | 0.989 |
| *Registry site, California vs. Non-California* | 0.144 | 1.079 | 0.974 | 1.196 |  | - |  |  |  |
| *T stage, reference: T_0-1_* | 1.000 |  |  |  |  | 1.000 |  |  |  |
| T*_2_* | < 0.001 | 1.513 | 1.291 | 1.773 |  | 0.002 | 1.518 | 1.294 | 1.781 |
| T*_3_* | < 0.001 | 2.546 | 2.150 | 3.015 |  | < 0.001 | 2.422 | 2.035 | 2.883 |
| T*_4_* | < 0.001 | 3.003 | 2.328 | 3.872 |  | < 0.001 | 3.048 | 2.353 | 3.948 |
| *N stage, Negative vs. Positive* | < 0.001 | 1.315 | 1.186 | 1.458 |  | < 0.001 | 1.246 | 1.119 | 1.387 |
| *EBRT, No/Unknown vs. Yes* | < 0.001 | 0.326 | 0.275 | 0.387 |  | < 0.001 | 0.559 | 0.455 | 0.685 |
| *CTx, No/Unknown vs. Yes* | < 0.001 | 0.319 | 0.279 | 0.365 |  | < 0.001 | 0.428 | 0.364 | 0.503 |

Abbreviations: W-F, Well or fairly differentiated; P-U, Poorly or undifferentiated; EBRT, external beam radiation therapy; CTx, chemotherapy; HR, hazard ratio; CI, confidence interval.
